# Overview of *Schistochilopsis* (Hepaticae) in Pacific Asia with the Description *Protochilopsis* *gen. nov.*

**DOI:** 10.3390/plants9070850

**Published:** 2020-07-06

**Authors:** Vadim A. Bakalin, Vladimir E. Fedosov, Yulia D. Maltseva, Irina A. Milyutina, Ksenia G. Klimova, Hung Manh Nguyen, Aleksey V. Troitsky

**Affiliations:** 1 Laboratory of Cryptogamic Biota, Botanical Garden-Institute FEB RAS, Makovskogo Street 142, 690024 Vladivostok, Russia; maltceva.yu.dm@gmail.com (Y.D.M.); ksenia.g.klimova@mail.ru (K.G.K.); 2 Faculty of Biology, Lomonosov Moscow State University, Leninskie Gory Str. 1–12, 119234 Moscow, Russia; fedosov_v@mail.ru; 3 Belozersky Institute of Physico-Chemical Biology, Lomonosov Moscow State University, Leninskie Gory 1, 119991 Moscow, Russia; iramilyutina@yandex.ru; 4 Institute of Ecology and Biological Resources, Vietnam Academy of Science and Technology, 18 Hoang Quoc Viet, Cau Giay, Hanoi 10000, Vietnam; nh.manhiebr@iebr.ac.vn

**Keywords:** *Schistochilopsis*, *Lophozia*, Scapaniaceae, East Asia, molecular phylogenetics, ITS rDNA, *trn*L–*trn*F, *trn*G

## Abstract

The taxonomic diversity center of *Schistochilopsis* is in East Asia, where the genus also shows the highest genetic diversity and morphological plasticity. The integrative survey of *Schistochilopsis* in East Asia was the main goal of the present account. Plant materials were obtained from recent collections made by authors in various parts of amphi-Pacific Asia; several types of specimens were also studied. The study includes phylogenetic reconstructions from nuclear ITS1,2, chloroplast *trn*L and *trn*G sequences, and anatomo-morphological, biogeographical, and taxonomical analyses. As a result, it was concluded that *S. obscura* should be transferred to *Lophozia* s. str. and *S. grandiretis* to the newly described genus *Protochilopsis*. *Lophozia boliviensis* was found to be part of the Andean–Sino-Himalayan taxon belonging to *Schistochilopsis*. The species status of *S. hyperarctica* and *S. opacifolia* was not confirmed. Substantial genetic variation is observed within *S. incisa* with possible cryptic sympatric distributed entities. The taxonomical section of the paper provides a discussion on the status, distinctive morphological traits, distribution, and ecology supplemented with the morphological description for poorly understood taxa. In the vast majority of cases, the illustrations and photographs made from the types and living material are provided.

## 1. Introduction

*Schistochilopsis* is not a speciose genus of hepatics. Söderström et al. [1] listed seven taxa, including *S. nakanishii* (Inoue) Konstant. for which there are serious doubts about its status. Two more taxa were added to the genus by Bakalin and Klimova [2]; thus, the formally recognized diversity was confined to nine taxa until present (http://www.catalogueoflife.org/annual-checklist/2019/). Three species (*S. grandiretis* (Lindb. ex Kaal.) Konstant., *S. incisa* (Schrad.) Konstant., and *S. opacifolia* (Culm. ex Meyl.) Konstant.) have nearly circumpolar distribution occurring from hemiarctic to hemiboreal zones, and then far southward in oroboreal belt in the mountains, though with a reported disjunction in South America for *S. incisa* [3,4]. Five taxa are known only in East Asia (*S. cornuta* (Steph.) Konstant., *S. nakanishii, S. pacifica* Bakalin, *S. obscura* Bakalin*, S. setosa* (Mitt.) Konstant.) and one (*S. hyperarctica* Konstant. & L. Söderstr.) is a rare taxon of presumable circum-arctic distribution. Therefore, East Asia should be regarded as the key region for the understanding of the diversity and probable speciation of *Schistochilopsis*. Taking this as the starting point, we tried to review this genus in East and North-East Asia based on the types and other available specimens.

### Historical Background

The history of currently recognized *Schistochilopsis* started from Müller [5], who first recognized the subgenus *Massula* within *Lophozia* s.l. (as the genus was accepted at that time). Müller [5] stressed numerous and small oil bodies as one of the valuable subgeneric characters. Currently recognized *Obtusifolium* and *Heterogemma* were included in the subgenus. Unfortunately, subg. *Massula* was an invalid name because it was not supplied with Latin diagnosis. Schuster [6] overlooked that problem of validity while exhaustively circumscribing this group under the name *Lophozia* subg. *Massula* Müller emend. Schuster, and provided many diagnostic characters valuable for the understanding of *Schistochilopsis* until the present, despite the type of subg. *Massula—Lophozia marchica* (Nees ex Limpr.) Steph. (= *Heterogemma capitata* (Hook.) Konstant. & Vilnet) now belongs to another genus. This misfortunate ‘nomenclatural’ start pursued the group of taxa for several decades. Schljakov [7] evaluated ‘subg. *Massula*’ as *Massula* (Müll.Frib.) Schljakov. This combination was invalid not only because it was the latter homonym of *Massula* Dulac (Typhaceae), but also because the genus *Massula* could not be founded on nomen nudum published by Müller [5].

Only eight years later, Schljakov [8] has found his combination was invalid due to the absence of Latin description in [5], and provided a new Latin description for *Massula* while publishing it as a new genus. Schljakov (l.c.), though, excluded *Obtusifolium* from *Massula* (following to [9]), nevertheless keeping the later recognized *Heterogemma* within *Massula* (*Heterogemma* was founded as a section within *Lophozia* s.l. based on *Lophozia mildeana* Gottsche (= *Heterogemma capitata*) by Jørgensen [10]). Later, Schljakov [11] found the homonymy of *Massula* Schljakov, as mentioned above. He then (l.c.) described *Massularia* Schljakov to replace invalid *Massula* Schljakov. Once again, it was a bad choice because it was the later homonym of *Massularia* (K. Schum.) Bullock ex Hoyle (Rubiaceae).

Kitagawa [12] accepted ‘*Lophozia* subg. *Massula*’ though regarded *Lophozia incisa* (Schrad.) Dumort. (= *Schistochilopsis incisa*) as the type of the subgenus that contrasts with Schuster [13], who lectotypified the subgenus by *Lophozia marchica*. Moreover, Kitagawa [12] overestimated the taxonomic importance of leaf keel wing in the *Lophozia* s.l. and described the new subgenus *Schistochilopsis* with *Lophozia cornuta* (Steph.) S. Hatt. (= *Schistochilopsis cornuta*) as the type. Bisang [4], in the revision of the “subgenus *Massula*”, with the estimated keel feature not being enough to maintain the different subgenera concept within the large genus *Lophozia* (as it has been still treated at that time), showing that *Lophozia* subg. *Schistochilopsis* is the legitimate name for the group in which both subg. *Massula* and subg. *Schistochilopsis* are included.

Konstantinova and Vasiljev [14] elevated *Lophozia* subg. *Schistochilopsis* N. Kitag. (sensu Bisang [4], but excluding *Obtusifolium*) to the rank of genus and created corresponding combinations within it. *Obtusifolium* was accepted as a separate genus, although was later re-included in *Schistochilopsis* [15]. The latter conclusion, based only on morphology, is hardly acceptable and not consistent with molecular data, according to which sect. *Heterogemma* has been excluded from *Schistochilopsis* as evaluated at the rank of genus [16,17,18] even been placed within another family (Lophoziaceae, versus Scapaniaceae, where *Schistochilopsis* was finally placed). The corresponding combinations were proposed by Konstantinova and Vilnet [19] for *Heterogemma laxa* (Lindb.) Konstant. & Vilnet and *H. capitata*. The third taxon, *Heterogemma patagonica* (Herzog et Grolle) L.Söderstr. et Váňa, based on *Lophozia patagonica* Herzog et Grolle, was later added to the genus [20].

Bakalin and Klimova [2] described two more taxa, *Schistochilopsis pacifica* and *S. obscura*, based on specimens from Pacific North-East Asia (Kamchatka and South Kurils). The phylogenetic relationships of both as well as their status were not tested genetically. Moreover, infrageneric relationships of taxa classified in *Schistochilopsis* also remained poorly known. There are ca. 40 *Schistochilopsis* sequences for various markers (*rbc*L, *nad*1, *psb*T–*psb*N–*psb*H, *rps*3, *rps*4–*trn*S, *trn*K–*psb*A–*trn*H, *trn*L–*trn*F*, trn*G, ITS) in GenBank, mostly used to reconstruct the high-level phylogeny of Hepaticae. Noticeable, among the aforementioned forty sequences, we found that only for three specimens were both ITS and *trn*L–*trn*F sequences represented in GenBank, and there are no specimens for which ITS–*trn*G and *trn*G+*trn*L–*trn*F pairs were found. Thus, for infrageneric research, a more thorough sequence dataset should be created.

## 2. Material and Methods

### 2.1. Study Area and Specimen Collection

The *Schistochilopsis* specimens were collected in North-East and East Asia. Plant sampling localities in the present study are spread from 73° N in Dikson Area, northwestern coast of Taimyr Peninsula to 20° N in Northwest Vietnam (Hoang Lien Range), but the vast majority of the involved specimens are from extra-tropical Pacific Asia, where the genus *Schistochilopsis* is the most diverse. Most of this area is under a monsoon climate with air mass circulation, while northward of 44° N, the climate in sampled areas varies from oceanic to continental. The vegetation is changed from Arctic tundra in the North to mountain subtropics in the South. This huge land was an arena for speciation at various taxonomic categories, and florogenetically, is tightly connected with Sino-Himalaya [21]. Although landscapes and biota of northeastern Asia (belonging to so-called Megaberingia, cf. [22,23]), have not been removed by glaciations during Cenozoic, they underwent crucial changes driven by Milankovich oscillations, resulting in extensive migration, speciation, and extinction events as well as further expansion to younger, cool temperate boreal and Arctic biomes [24].

The basic information on climatic parameters near collection localities is in Table 1. These data provide very general information of the climates in the places of specimen collection because all of the listed localities are characterized by highly mountainous relief, and although we selected cities nearest to the collecting localities, the altitudinal variations are surely neglected despite perhaps strongly affecting the actual climate characteristics in the sites of collection.

### 2.2. DNA Isolation and Sequencing

The DNA was extracted from dried liverwort tissue using the NucleoSpin Plant Kit (MachereyNagel, Germany). Amplification of ITS1–5.8 S–ITS2 nrDNA, *trn*L–*trn*F and *trn*G intron cpDNA was performed using Encyclo Plus PCR kit (Evrogen, Moscow, Russia) and primers listed in Table 2.

PCR was carried out in 20 μL as follows: initial denaturation at 95 °C for 3 min; followed by 30 cycles of denaturation at 94 °C for 30 s, annealing at 58–62 °C for 30 s, elongation at 72 °C for 1 min; and final extension at 72 °C for 3 min.

DNA was sequenced using the BigDye Terminator v. 3.1 Cycle Sequencing Kit (Applied Biosystems, USA) with further analysis of the reaction products on the ABI Prism 3100-Avant Genetic Analyzer (Applied Biosystems, USA) in the Genome Center (Engelhardt Institute of Molecular Biology, Russian Academy of Sciences, Moscow). The list of determined sequences and their GenBank accession numbers are indicated in Table 3.

### 2.3. Phylogenetic Analysis

For molecular phylogenetic study, three markers, nuclear ITS1,2 and 5.8 rRNA gene, and plastid *trn*L–F region and *trn*G gene, were successfully used in a number of previously published phylogenetic reconstructions of Hepaticae phylogeny. The laboratory protocol was essentially the same as in previous moss studies, described in detail by, e.g., Gardiner et al. [31], Hedenäs [32], and Bakalin and Vilnet [33]. Sequences were aligned using MAFFT v. 7.402 [34] standard settings and then adjusted manually in BioEdit [35]. For phylogenetic reconstructions, three datasets were used: ITS1–5.8S rDNA–ITS2 (47 accessions, 825 positions), *trn*L–*trn*F+*trn*G (42 accessions, 1310 positions), ITS1–5.8S rDNA–ITS2+ *trn*L–*trn*F (36 accessions, 1347 positions), and *trn*G (71 accessions, 783 positions). Bayesian analyses were performed by running two parallel analyses in MrBayes 3.2.7a [36] using the GTR+I+G model. For single gene set analyses, each run consisted of six Markov chains, 10,000,000 generations with a default number of swaps, and sampling frequency of one tree every 2500 generations. For the combined datasets, the analysis consisted of eight Markov chains and 25,000,000 generations; with the default number of swaps and sampling frequency, one tree each 5000 generations was performed. The chain temperature was set at 0.02 in all analyses. Consensus trees were calculated after discarding the first 25% trees as burn-in. While analyzing the *trn*L–*trn*F set, indels were coded using simple indel coding approach [37] in SeqState 1.4.1 [38]. Analyses were performed on the Cipres Science Gateway [39]. Within-group and between-group mean *p*-distances were calculated using MegaX [40].

The secondary structure modeling of *trn*G intron was performed by RNAstructure 6.2 [41] and jViz.Rna 4.0 visualization tool [42].

Additionally, a large set of specimens was analyzed morphologically, also including the types of *Schistochilopsis cornuta, S. nakanishii, S. setosa,* and *Lophozia boliviensis* that were too old to be sequenced. All studied (for the first time or revised) specimen sequences were deposited into and are available online at the database maintained by VBGI (http://botsad.ru/en/herbarium/) with the morphological concept used for delimitations within the *Schistochilopsis incisa* complex that is partly not supported by the present results. However, we retained the use *S. opacifolia* for the specimens we were not able to test genetically.

## 3. Results

**Figure 1:**
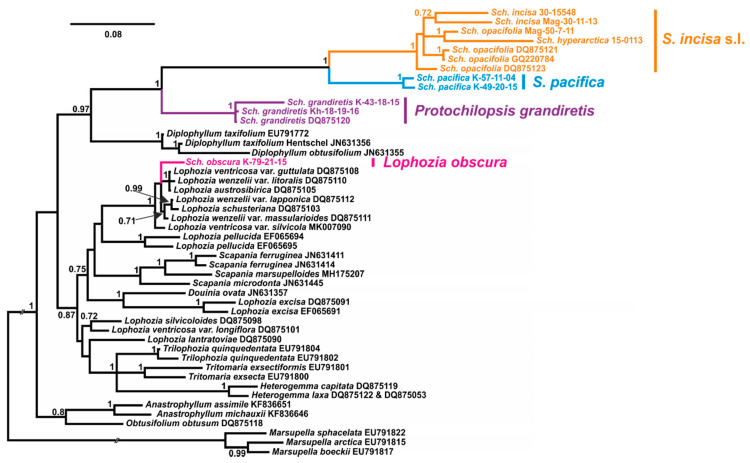
Bayesian tree of ITS1–5.8S–ITS2 sequences. *Sch*.—*Schistochilopsis.* Specimen titles (according to Table 3) or GenBank accession numbers are given after the species names. Posterior probability values >0.5 are indicated. Scale bar denotes number of nucleotide substitutions per site.

**Figure 2:**
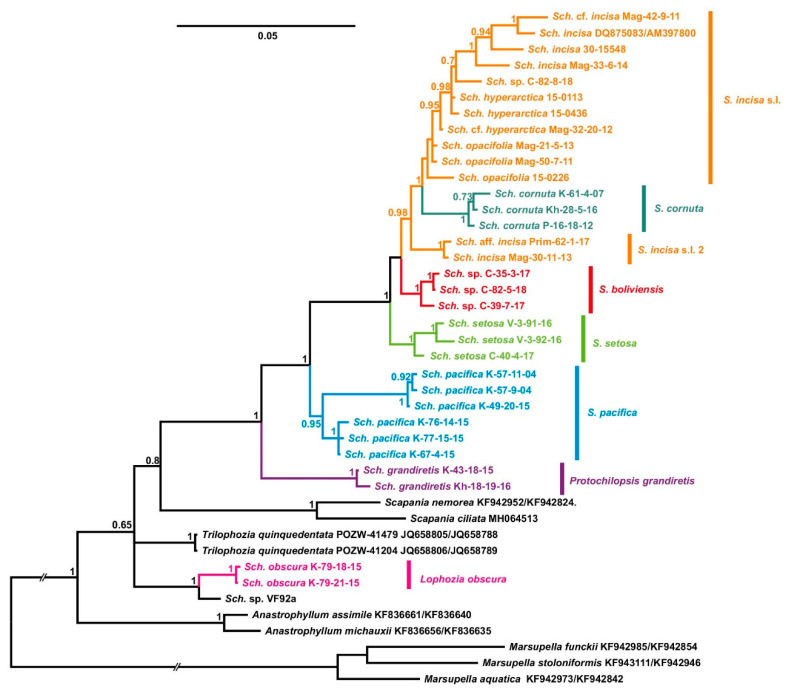
Bayesian tree of t*rn*L–*trn*F+*trn*G sequences. *Sch*.—*Schistochilopsis.* Specimen titles (according to Table 3) or GenBank accession numbers are given after the species names. Posterior probability values >0.5 are indicated. Scale bar denotes number of nucleotide substitutions per site.

**Figure 3:**
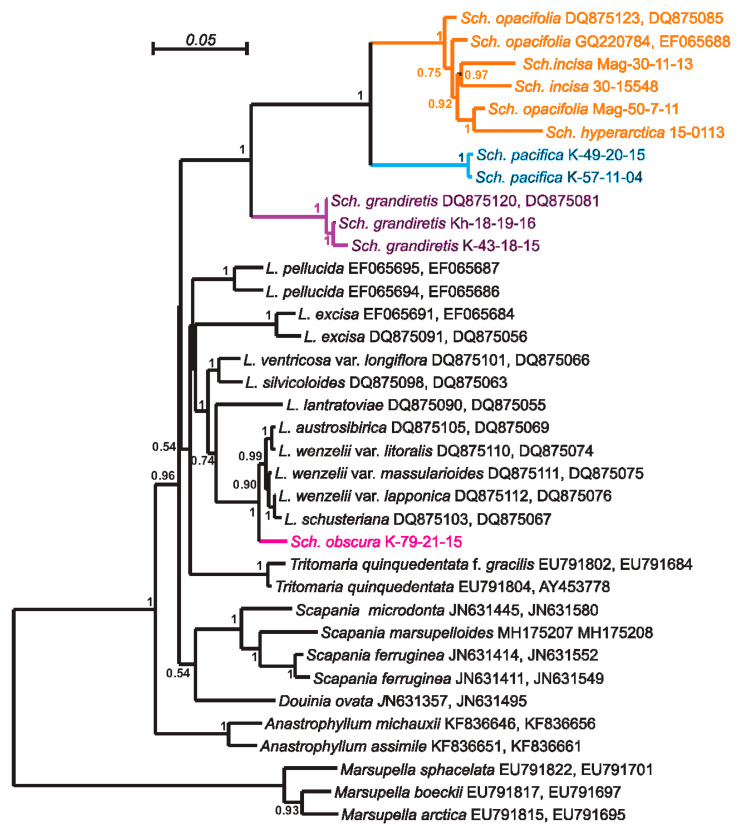
Bayesian tree of ITS+t*rn*L–*trn*F sequences. Posterior probability values >0.5 are indicated. *Sch*.—*Schistochilopsis, L.*—*Lophozia.* Specimen titles (according to Table 3) or GenBank accession numbers are given after the species names. Posterior probability values >0.5 are indicated. Scale bar denotes number of nucleotide substitutions per site.

**Figure 4:**
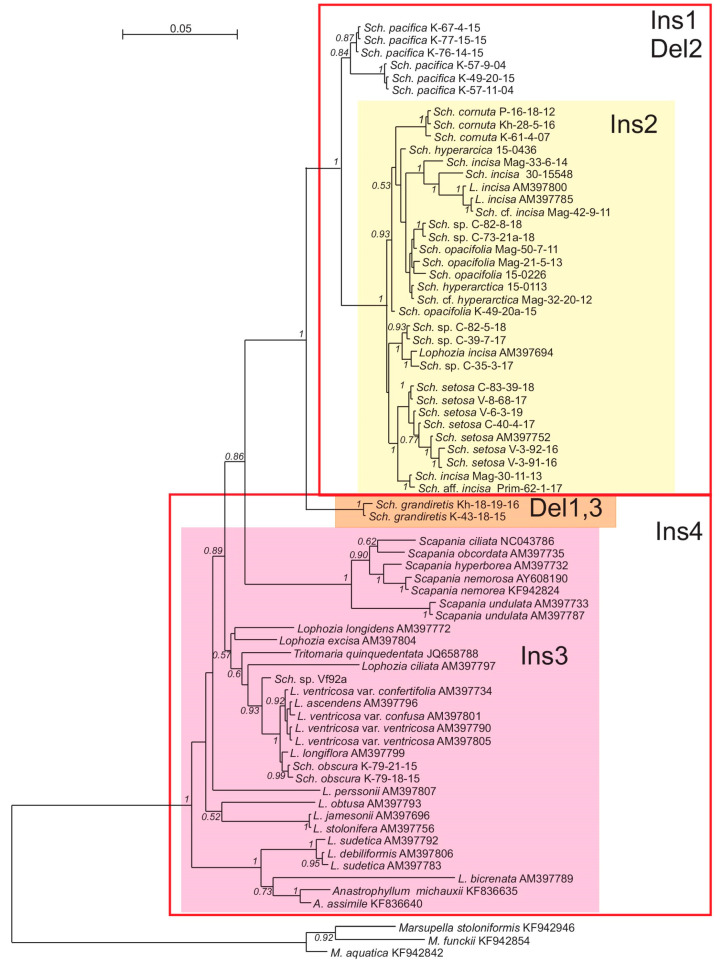
Bayesian tree of *trn*G sequences. Posterior probability values >0.5 are indicated. *Sch*.—*Schistochilopsis, L.*—*Lophozia.* Specimen titles (according to Table 3) or GenBank accession numbers are given after the species names. Posterior probability values >0.5 are indicated. Scale bar denotes number of nucleotide substitutions per site. Ins—insertions, Del—deletions in the sequences.

Despite the sufficient difference in the composition of datasets, the resulting tree topologies were congruent (Figure 1, Figure 2, Figure 3 and Figure 4). The vast majority of specimens, assigned to *Schistochilopsis* (excepting those of *S. obscura*), form a well-supported clade sister to the *Diplophyllum* clade in the ITS-based reconstruction, and the *Scapania* clade in the plastid trees (Figure 2 and Figure 4) or to *Lophozia*+*Tritomaria* in ITS+*trn*L–*trn*F tree (Figure 3).

Accessions of *Schistochilopsis obscura* form a well-supported clade with the single *Lophozia* specimen in the *trn*L–*trn*F+ *trn*G tree (Figure 2) or nested in the well-supported *Lophozia* clade in the ITS, ITS+ *trn*L–*trn*F, and *trn*G-based trees (Figure 1, Figure 3 and Figure 4).

**Figure 5:**
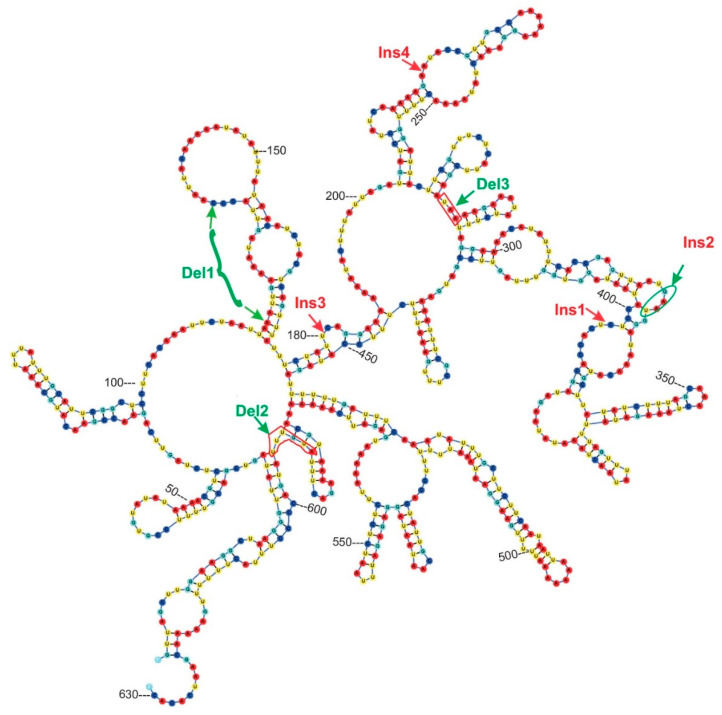
Secondary structure of *trn*G intron from *Schistochilopsis* sp. (sichuanica) C-39-7-17. Positions of group-specific indels along the phylogenetic tree on Figure 4 are indicated.

In all cases, the maximally supported *S. grandiretis* clade occupies a sister position to the maximally supported and well-differentiated clade formed by all other *Schistochilopsis* specimens except *S. obscura*. Comparison of inter- and infragroup *p*-distances additionally indicates a distinct position of *S. grandiretis* among *Schistochilopsis* species. The between-group *p*-distance (*S. grandiretis* clade versus “core *Schistochilopsis* clade”) accessed for combined *trn*L–*trn*F + *trn*G dataset is 0.062, while the within-group mean distance in the “core *Schistochilopsis* clade” is ca. 0.03. Likewise, between-group distance inferred from the ITS alignment is 0.23, while within-group mean distance for the core *Schistochilopsis* clade is ca. 0.08. *trn*G intron sequences of studied hepatics possessed group-specific indels, which are marked in Figure 4 and indicated in the intron secondary structure model in Figure 5. As indicated in Figure 4, indels separate *S. grandiretis* from the rest of the *Schistochilopsis* species and bring it together with other hepatics on the tree.

A basal position among *Schistochilopsis* species, except for *S. grandiretis*, was occupied by *S. pacifica* accessions divided into two well-supported clades in Figure 2 and Figure 4 followed by *S. incisa* s.l. 2, *S. setosa*, *S. bolivensis*, and a clade designated as *S. incisa* s.l. 1 of intermixed specimens of *S. opacifolia, S. hyperarctica*, and *S. incisa* with nested *S. cornuta* clade.

Despite the presence of well-supported nodes in *S. incisa* s.l. 1 clade, the pattern of grouping within it reflects neither present concepts of these species nor any other morphological or geographical trend.

## 4. Discussion

The new insights into the taxonomy of species currently considered within the genus *Schistochilopsis* as inferred from the obtained phylogenetic reconstructions and herbarium studies and may have resulted from the following suggestions:

### 4.1. Position of Schistochilopsis obscura

*Schistochilopsis obscura* should be transferred to *Lophozia* s. str. providing the corresponding combination. This species probably (available data are somewhat incomplete) occupies the basal position in the latter genus. The morphological similarity of *Schistochilopsis obscura* and *Lophozia* was stressed at the original description when authors [2] were obliged to choose which morphological feature is more valuable for the systematics: the presence of microcellous layer in the stem cross section or stable leaf bilobation (trilobed leaves occurred only as rare exceptions, and commonly occur in other *Lophozia* s. str. taxa). However, the taxonomic value of the definition of ‘stable bilobation’ is hardly tenable also because many modifications of *Schistochilopsis incisa* and the vast majority of *S. cornuta* accessions populations have mostly bilobed leaves (thus, the same as for *Lophozia* s. str.). Due to the aforementioned, authors of [2] estimated the absence of a microcellous layer as the basic feature and placed the species into *Schistochilopsis*. However, this point of view was incorrect as demonstrated by the present molecular data. Therefore, *S. obscura* is the first species of *Lophozia* s. str. in which the microcellous layer is not observed in all available materials. It is worth mentioning that the microcellous layer commonly becomes thinner in several *Lophozia* taxa in their southern localities, although even there (e.g., *L. pallida* in the southern China or *Lophozia ventricosa* s.l. in the Caucasus), they never became completely extinct. The only example of a taxon without a microcellous layer is *Lophozia wenzelii* var. *massularioides* Bakalin, whose status (and phylogenetic relationships) remains unclear. On the other hand, the microcellous layer is commonly completely absent in laboratory-cultured *Lophozia* [43].

Taking into account the complicate recent geological history of the Greater Kurils (including Iturup Island, where *Schistochilopsis obscura* was collected) and the location of the habitat in the volcanically active area (the late-Pleistocene caldera now partly filled by the strato-volcanic cone: http://www.kscnet.ru/ivs/lggp/ot2001/r211.htm) we cannot regard this species as local endemic. Since *Schistochilopsis obscura* has abundant gemmae, we may suggest its wider distribution across Kurils and possibly in Hokkaido Island than it is presently known. However, when working in Kunashir Island in 2018, we did not find it despite a purposeful search.

### 4.2. Position of Schistochilopsis grandiretis

The position of *Schistochilopsis grandiretis* is basal and highly distanced from other *Schistochilopsis* taxa. Moreover, the morphology of *S. grandiretis* is quite far from the bulk of *Schistochilopsis*. The species is characterized by exceedingly large midleaf cells averaging 50–80 µm × 40–60 µm, invariable purple-black pigmentation in the ventral side of the stem, and the common presence of purple pigmentation in leaves, especially near shoot apices. These features do not occur in other taxa of *Schistochilopsis* and show superficial similarity with *Heterogemma laxa* (morphologically different, however, in the ellipsoidal gemmae and distanced 2–3-lobed leaves). The rather high distance separated *S. grandiretis* from the rest of *Schistochilopsis*, supported by analyses of group-specific indels in the *trn*G intron, and suggests this species should be separated into a monospecific new genus that is described in the taxonomical section below.

### 4.3. Position of Schistochilopsis pacifica

After transferring *Schistochilopsis grandiretis* from *Schistochilopsis* to its own genus, the basal group in *Schistochilopsis* in all trees was occupied by the recently described *Schistochilopsis pacifica* (Figure 1, Figure 2, Figure 3 and Figure 4). The latter species is characterized, in comparison with other taxa, by relatively orthotropic growth, presence of golden-brownish pigmentation in the apical part of apical leaves (like reported for *S. hyperarctica*), less obliquely inserted leaves, and lesser-developed leaf dentation. These features may probably suggest orthotropic growths and the ability to develop brownish pigmentation (widely distributed in Scapaniaceae as the whole) as the plesiomorphic features. The poor dentation along the leaf margin may also be a plesiomorphic feature in the genus. Schuster [6] (p. 86) estimated the specialization within *Schistochilopsis* (*Lophozia* subg. *Massula* in l.c.) in the following way: “cells become non collenchymatous both in leaves and stems; stem cells become narrow and elongate; oil bodies become minute and numerous; leaves retain ability to form 4–5-lobes; ventral stem sectors may stay broad”. This trend somewhat contradicts to the obtained trees topologies. *Schistochilopsis pacifica* has a ventral segment sometimes narrower than in the *S. incisa-opacifolia* complex and reaching only 3–5 cells broad. The leaf cells are more leptodermous in *S. pacifica* than in xeric modifications of *S. setosa* and *S. incisa*. The oil body features are hard to estimate because their numbers per leaf cell greatly vary within each taxon (although always remain relatively high).

*Schistochilopsis pacifica* shows the substantial genetic variation in areas not so far in distance: southern part of East Kamchatka (from where the type) and southern Kurils (Iturup, from where the paratypes), with the only 8° between the localities. The genetic distances permit the description of two subspecies within *S. pacifica* (Figure 2 and Figure 4), but we refrain from this because there is no satisfactory morphological base for it. The specimens from the South Kurils (Table 3) contain plants with more densely dentate leaves and more green color, but these features may be environmentally induced. They could hardly be accepted for taxonomic differentiation of two putative subspecies.

### 4.4. Status of Schistochilopsis hyperarctica

The species status of *Schistochilopsis hyperarctica* is not confirmed. We are inclined to treat it as the extreme modification of widely distributed and morphologically variable *S. incisa* s.l., although some morphological features (the presence of brownish pigmentation, predominantly bilobed leaves) may suggest the species status. The only reason we refrain from providing the formal synonymization of *S. hyperarctica* with *S. incisa* is that we did not involve the specimens tentatively named as *S. hyperarctica* collected in Eastern Canadian High Arctic—the place from where *S. hyperarctica* was described. However, we may argue, with a certain confidence, that the specimens originally named as *S. hyperarctica* in Siberian Arctic and North-East Asia actually belong to the morphologically malleable *S. incisa* s.l.

### 4.5. Circumscription of Schistochilopsis grandiretis

*Schistochilopsis setosa* was found to be morphologically malleable. The plants from Yunnan in China and Northwest Vietnam are quite different in morphology: plagiotropic growth and dense dentation in leaf margins in Hengduan Range versus orthotropic growth and less dentate leaves in specimens from Hoang Lien Range. However, genetically, this is the single species restricted by Eastern Sino-Himalaya and mountainous Northern Indochina (the specimens from western Himalaya were not tested genetically). The features of the species include 3–5-lobed leaves that, commonly, are prominently toothed, with the end cell in the tooth usually exceeding 100 µm long. The species was observed in forested areas of highest altitudes in Northwest Vietnam (there are no forestless alpine landscapes in Indochina), where it has a lax texture with not so prominent teeth, to the base unistratose and commonly distanced leaves. These features are in contrast to those in plants from Sichuan and Yunnan collected above timberline or in crooked forests and possess densely contiguous leaves with margins densely and prominently dentate, such they commonly look as though they are covered by large crystals due to colorless, glistening, and numerous teeth (the same may be said for the type of the taxon that is from Indian Sikkim). Additionally, the plants in Chinese specimens are commonly deep green colored, whereas Vietnamese plants are duller. As far as it was found in the genetic analysis, the listed differences are the only environmentally induced features.

### 4.6. Position of ‘Lophozia boliviensis’

One more morphologically malleable taxon should be revisited. We initially revealed the possible new taxon based on the collections from West Sichuan and named it as a potentially new species. The basic estimated features include subentire, 2–3-lobed, subtransversely inserted leaves. Later, when the material from Northern Yunnan was included in the analysis, the concept of the species underwent serious modifications, since it was found (1) the leaves are sometimes dentate, although not so prominently as in *S. setosa*, (2) the outer cell wall in the leaf is usually very thick and additionally brown colored, (3) the cell size in the midleaf is far smaller than that along the margin, as well as compared to *S. setosa*. These features, as found in the molecular study, are correlated with genetic differences between the plants that could not be referred to as *S. setosa*.

The most unexpected result was found when the GenBank accessions for *Schistochilopsis* were added to the dataset. The specimen named as *Lophozia incisa* (AM397694), collected by Söderström in Venezuela, found by *trn*G sequence, was very similar to the accessions from Sichuan (Figure 3). Indeed, there was the data on supposed *Schistochilopsis incisa* occurrence in the Andean Mountains. However, initially and as early as Stephani described Herzog’s collection of *Schistochilopsis* from Bolivia as *Lophozia boliviensis* Steph. (G00061164/1651!)., Schuster (*in litt.*) wrote on the label only “*Lophozia* (subg. *Massula*) *boliviensis* St. type”. By contrast, Vana firstly questioned its conspecific nature with “*Lophozia incisa*” in the additional label inscription in G (Herbarium of the Conservatoire et Jardin botaniques de la Ville de Genève), *in litt*., but later treated “*Lophozia boliviensis*” as an undoubted synonym of *L. incisa* (= *Schistochilopsis incisa*), e.g., in [3]. However, in the course of study the type of “*Lophozia boliviensis*” in G, that is small and poorly preserved (cells are mostly collapsed inward of the leaf, whereas the only well-developed cells are along female bract margins), we found this is the same with aforementioned specimens from Sichuan, or, at least, highly similar. *Lophozia boliviensis* does not have prominently toothed leaves (sterile leaves are almost not toothed at all), and a thick marginal wall of the leaf is subtransversely inserted, although obliquely oriented leaves. Two consequences are therefore possible: (1) the *Lophozia boliviensis* type, the Venezuelan specimens AM397694 collected by Söderström, and the materials from Sichuan belong to the same taxon and a new combination for *Lophozia boliviensis* is necessary, (2) the distribution of *Schistochilopsis incisa* in Andean Mountains should be doubted, and all reports of the species from there may actually belong to Himalayan-Andean disjunct relict taxon (the occurrence in Kilimanjaro in Africa may also be expected).

Interestingly, *Schistochilopsis setosa* and ‘*Lophozia boliviensis*’ have occurred in the same locality in Yunnan although never intermixed. Moreover, the third species was once found in Yunnan (C-82-8-18)—*Schistochilopsis incisa* that, however, distinctly differs from *‘Lophozia boliviensis’* in not having thickened external cell wall in leaves and 2–4-lobed obliquely inserted leaves.

### 4.7. Position of Schistochilopsis cornuta

*Schistochilopsis cornuta* is a well-delimited hemiboreal to temperate East Asian amphi-Pacific taxon. Its position in phylogenetic trees on Figure 2; Figure 3 does not require the recognition of separate subgenus cf. [12], as shown before by Bisang [4] based on morphological evidence. The main morphological feature of this taxon—leaf wing—is very variable in size to its almost complete absence. The wing width also varies across the distribution area and seems to be larger in the southern part of the area.

### 4.8. Circumscription of Schistochilopsis incisa s.l.

The group *Schistochilopsis incisa*–*S. opacifolia*–*S. hyperarctica* is still unresolved, both genetically and morphologically. *Lophozia opacifolia* Culm. ex Meyl. (= *Schistochilopsis opacifolia*) was revealed by Culmann [44,45], although even at the time of description, the author doubted the status of the taxon (cf. [13] (p. 452)). Schuster [13], nevertheless, recognized it at the species level as different from *S. incisa* in opaque plants, ‘nonspinose’ and tending to have 3–5-stratose leaves in the base and additionally characterized by broader, not so acute leaf lobes, subentire to denticulate perianth mouth and larger spores. At the same time, Schuster [13] (p. 455) stressed that “admittedly, transitional forms between the two species occur”. The Schuster’s [13] point of view was widely accepted, although later, he reduced the rank of the taxon to subspecies (*Lophozia incisa* subsp. *opacifolia* (Culm. ex Meyl.) R.M. Schust. & Damsh.) [46]. The latter point of view was accepted by Bisang, who also did not maintain the species status for *S. opacifolia* based on morphological studies (although noted the number of distinguishing morphological features) [4]. Then, Bakalin [47] followed the points of view of Schuster [46] and Bisang [4] and accepted the subspecies status for *Schistochilopsis opacifolia*. Noteworthy, Bakalin’s concept was based primarily on molecular data available at that time, namely, the genetic distance between some populations of ‘*S. opacifolia*’ is higher than between populations of *S. incisa* and certain populations of *S. opacifolia* (cf. [47]).

Within recent years, the species status for *Schistochilopsis opacifolia* was accepted in the World liverwort checklist by Söderström et al. [1]. Bakalin and Klimova [2] allowed the species status of this taxon, too; it was made based on the suggestion that the robust distances between some populations in ‘*S. opacifiola*” is the result of inclusion of several undescribed taxa into this bulk, and therefore it is better to recognize *S. opacifolia* separately from *S. incisa*. In the course of the present work, we found all sequences designated as *S. opacifolia*, *S. incisa*, and *S. hyperarctica* are deeply intermixed in the phylogenetic trees (Figure 1, Figure 2, Figure 3 and Figure 4). Therefore, the possible explanations may be that the observable morphological differences are only environmentally induced and result from spatial isolation. When certain populations of single taxon survived in similar environments for a long time, they stochastically accumulated many specific features in the nucleotide sequences and then again formed widely overlapping areas.

### 4.9. Geographic Distribution of Schistochilopsis

The highest taxonomic diversity of *Schistochilopsis* in Pacific Asia is known in the areas with monsoon to oceanic hemiboreal to cool temperate climates in the mountains (data provided in Table 1). Widely distributed and eurytopic *Schistochilopsis incisa* s.l. stretches from the area of the northern extremes of the land on Asia to 30° N in the Sichuan Province of China. This gradient is characterized by negative winter temperatures and, in the northern extreme, also negative annual temperature (e.g., −11 °C in the north part of Magadan Province). Other taxa possess narrower amplitude. *Schistochilopsis pacifica* is distributed in the areas with strictly oceanic climates, with slightly positive annual temperatures, low (below 20 °C) temperatures for the warmest months, and slightly negative temperatures for the coldest month (−6 to −8 °C), associated with very thick snow cover. The annual amount of precipitation should be above 1000 mm per year (quite a high value for hemiarctic and boreal climates). *Schistochilopsis cornuta* is distributed in areas with the monsoon to oceanic environments in hemiboreal and temperate zones, commonly in mountainous areas. In all cases, the annual temperatures are positive, the coldest month negative (above −12 °C), and the yearly precipitation varies from 700 to 1200 mm or higher. *Schistochilopsis setosa* has a broad amplitude of annual temperature (at least from 5 to 16 °C) and winter temperatures (in January) varying from slightly negative in the Sichuan Province of China to distinctly positive in Northwest Vietnam. According to to data in hand, the amount of precipitation ranges from 800 to above 2000 mm per year, but always with a meager amount of rainfall in the coldest month (below 10 mm for the coldest month). The enigmatic ‘*Lophozia boliviensis*’ in East Asia grows in a monsoon climate with annual rainfall from 800 to 1000 mm per year, with the coldest month quite dry and the temperatures only slightly below or slightly above zero.

## 5. Taxonomic Treatment

***Schistochilopsis*** (N.Kitag.) Konstant., Arctoa 3: 125, 1994.

= *Lophozia* subg. *Schistochilopsis* N.Kitag., J. Hattori Bot. Lab. 28: 289, 1965.

Type species: *Schistochilopsis cornuta* (Steph.) Konstant. (= *Schistochila cornuta* Steph.)

The genus is characterized by:

1. Fleshy and soft plants, without red or purple pigmentation, even as traces.
2. Very wide (up to 8–10 cells in width) ventral segment of the stem that is however free of underleaves;
3. Instability of leaf thickness in the basal portions (2–3(5)-stratose leaf base is observable in some taxa);
4. Unstable lobation of the leaves, although 3-lobed leaves are dominant.
5. Leaf cells with numerous granulate (to coarsely so) oil bodies that commonly are biconcentric.

The genus includes seven species (one with doubtful status) occurring exclusively in the Holarctic with the main diversity center in East Asia. Below, we provide the taxonomic arrangements of *Schistochilopsis*, containing the descriptions for still poorly known taxa based on available materials.

***Schistochilopsis boliviensis*** (Steph. in Hezog) Bakalin et Fedosov ***comb. nov*.**

Basionym: *Lophozia boliviensis* Steph. in Hezog, Bibliotheca Botanica 87: 187, f. 103, 1916.

Lectotype (selected here, basing on Schuster, *in litt*.): Bolivia, 3700 m alt., 1912, Herzog, G00061164/1651!

Description based on the specimens involved in the present study (Table 3 and aforementioned holotype): plants prostrate to ascending, deep green to yellowish greenish, in loose patches, 1.2–1.8 mm wide, 2–10 mm long, deep green to greenish, in older (died) parts slightly brownish, but secondary pigmentation in living sectors is absent. Rhizoids numerous, brownish, in loose erect spreading fascicles, attaching plants to the substrate or other plants. Stem branching has seen only as 1–2 subfloral innovations; cross section transversely elliptic, ca. 250 µm × 450 µm, irregularly infested by fungal hyphae across the cross section (always absent in outer cells), ventral 2–3 cell layers brown colored, external walls of outer cells thickened, outer cells 17–30 µm, inward 25–38 µm, very thin-walled, trigones virtually absent. Leaves distant to contiguous and dense, overlapping to 2/3 of below situated leaf, obliquely to subtransversely inserted, oriented and spreading, not decurrent, concave-canaliculate or slightly recurved in upper half, when flattened in the slide nearly subquadrate, divided by V- to U-shaped sinus into 2–3(4) lobes, with sinus descending to 1/4–1/3(2/5) of leaf length, lobes equal in size or nearly so, 0.5–0.8 mm × 0.6–1.0 mm, margin entire to dentate, lobes acute, somewhat diverging to straight. Midleaf cells oblong, 25–45 µm × 17–35 µm, thin-walled, trigones small, concave to slightly convex, cuticle smooth; cells along leaf margin oblong along the margin, 30–55 µm, external wall thin to thick, trigones small, concave; margin entire to sparsely or densely dentate, teeth 1–2 cells long and 1–2 cells wide in the base, the apical tooth cell less 100 µm long, the basal part of the leaf not or very shortly toothed. Gemmae relatively small, 4–8-gonal, with no prominent angles, 2(–3)-celled, ca 20 µm in diameter or 25–30 µm × 20–30 µm. Dioicous. Only unfertilized and presumable undeveloped perianths are seen, those are obovate, with upper part curved downward, mouth crenulate-dentate; female bracts similar to leaves or larger, ob-trapezoidal, erect spreading.

**Figure 6:**
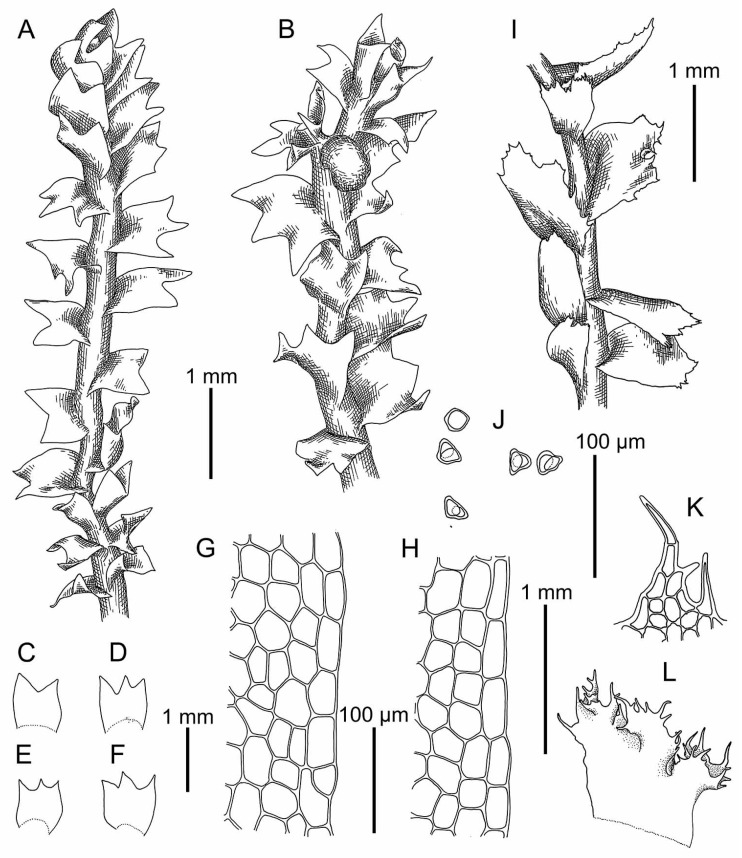
*Schistochilopsis boliviensis* (Steph. in Hezog) Bakalin et Fedosov: (**A**,**I**) Plant habit, fragment, dorsal view; (**B**) Plant with abnormal perianth, fragment, dorsal view; (**C**–**F**) Leaves; (**G**,**H**) Leaf margin cells; (**J**) Gemmae; (**K**) Cells of leaf apices; (**L**) Female bract. (**A**–**H**) from C-39-7-17 (VBGI); (**I**–**L**) from Holotype G00061164/1651.

**Figure 7:**
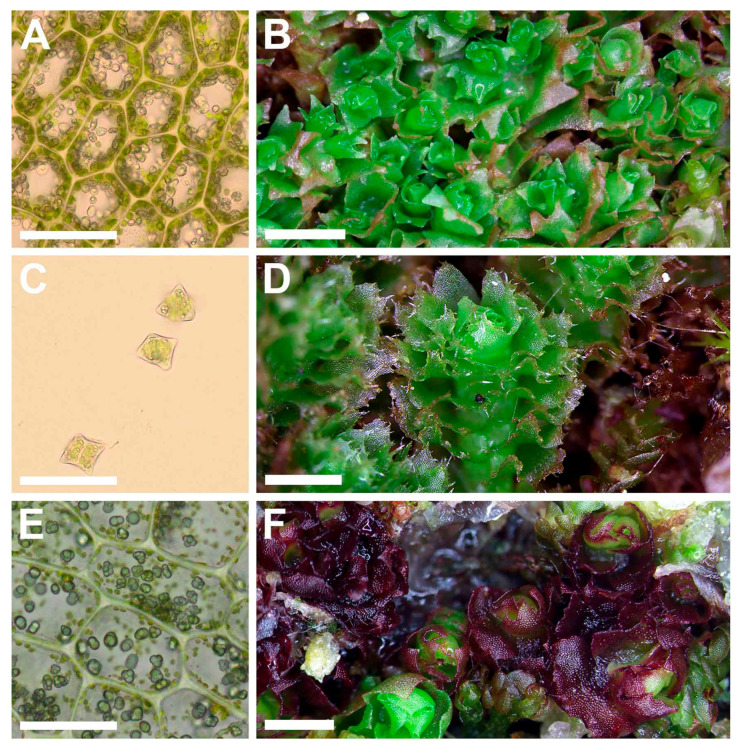
(**A**–**D**) *Schistochilopsis boliviensis* (Steph. in Hezog) Bakalin et Fedosov; (**E**,**F**) *Protochilopsis grandiretis* Troizk., Bakalin et Fedosov. (**A**,**E**) Leaf middle cells with oil bodies; (**C**) Gemmae; (**B**,**D**,**F**) Parts of mats. Scales: 100 µm for (**A**,**C**,**E**); 1 mm for (**B**,**D**,**F**). (**A**–**C**) from C-39-7-17; (**D**) from C-35-3-17; (**E**) from Mag-21-1-14; (**F**) from Khab-38-1-19, all from VBGI.

Illustrations in the present paper: Figure 6 and Figure 7A–D.

The distribution of this taxon looks enigmatic: Andean–Sino-Himalayan taxon, occurring in upper elevations of the mountains in orohemiboreal–orotemperate belts. Its real distribution may be broader, and the species may be found in high mountains of equatorial or subequatorial Africa (e.g., Kilimanjaro Mt.), from where, however, no records of *Schistochilopsis* exist.

The diagnostic features of the species include:

1. Polymorphic leaves varying from shortly to deeply 2–3-lobed (4-lobed only as exclusion);
2. Leaves are obliquely to subtransversely inserted, with margin ranging from dentate to entire, if dentate, then apical tooth cell less 100 µm long (commonly less 80 µm) and teeth become noticeably shorter to the leaf base.
3. Merely thin to very thick-walled external cell wall along the margin of dentate leaves (up to 6–7 µm thick), leaf margin cells are distinctly larger than cells inward.
4. Colorless gemmae, 2(–3)-celled, that are angular, with not strongly prominent angles.
5. Midleaf cells not large and commonly moderate in size to small and concave trigones.

The species is morphologically similar to *S. setosa* (with which it is distributed sympatrically in Hengduan Range) in sometimes thickened leaf margin cells, and obliquely to subtransversely inserted leaves. *Schistochilopsis boliviensis*, however, differs from *S. setosa* in marginal leaf cells larger than cells in the midleaf (versus midleaf cells larger than marginal in *S. setosa*), midleaf cells with commonly small and concave trigones (versus trigones moderate in size to large and commonly convex) and, if present, shorter tooth apical cell (less 100 µm versus mostly longer 120 µm). Moreover, leaves in the species are predominantly 2–3-lobed, not 3–5-lobed, as commonly occurs in *S. setosa*.

As was noted by Bisang [4], Herzog collected *Lophozia boliviensis* during his Bolivia trip; therefore, the number on the label in G (“1912”) may not be the collection date, or it may be a typo. ‘*Lophozia boliviensis*’ was typified by Schuster in 1964 but does not seem to be published anywhere.

***Schistochilopsiscornuta*** (Steph.) Konstant., Arctoa 3: 125, 1994.

Basionym: *Schistochila cornuta* Steph., Sp. Hepat. 4: 84, 1909.

Holotype: Japan, Jimba, October 1905, U. Faurie, no. 1879, G00061160!

Descriptions in [12] (p. 290).

**Figure 8:**
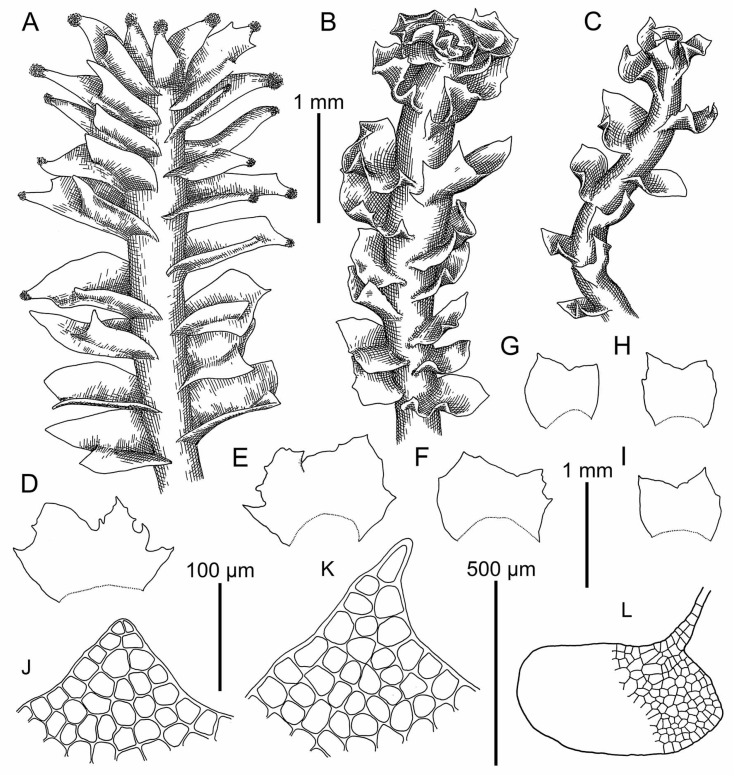
*Schistochilopsis cornuta* (Steph.) Konstant.: (**A**) Plant habit (on leaf apices gemmae in masses), fragment, dorsal view. *Schistochilopsis incisa* (Schrad.) Konstant.: (**B**,**C**) Plant habit, fragment, dorsal view; (**D**–**I**) Leaves; (**J**,**K**) Cells of leaf apices; (**L**) Stem cross section with leaf base. (**A**) from Holotype G00061160; (**B**–**L**) from Mag-30-11-13 (VBGI).

**Figure 9:**
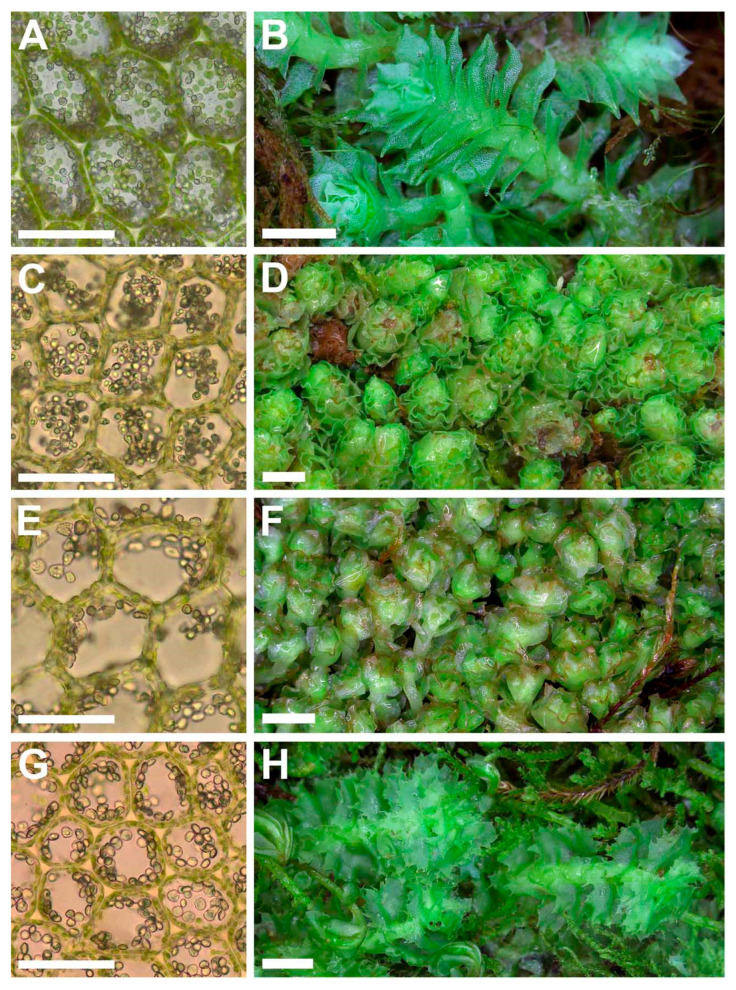
(**A**,**B**) *Schistochilopsis cornuta* (Steph.) Konstant.; *Schistochilopsis incisa* (Schrad.) Konstant. (**C**,**D**); *Schistochilopsis pacifica* Bakalin (**E**,**F**); *Schistochilopsis setosa* (Mitt.) Konstant. (**G**,**H**). Leaf middle cells with oil bodies (**A**,**C**,**E**,**G**), parts of mats (**B**,**D**,**F**,**H**). Scales: 100 µm for (**A**,**C**,**E**,**G**); 1 mm for (**B**,**H**); 2 mm for (**D**,**F**). (**A**) from P-36-19-14; (**B**) from J-8-20-15; (**C**,**D**) from K-49-20a-15; (**E**,**F**) from K-49-20-15; (**G**) from V-6-3-19; (**H**) from V-8-63-17, all from VBGI.

Illustrations in [12] (Figure XVI); the present paper: Figure 8A and Figure 9A,B.

Chiefly orohemiboreal–orotemperate Pacific Asian, occurring in the southern part of the Russian Far East, Korean Peninsula, Japan (throughout, but except Volcano, Bonin, and Ryukyu Islands), Taiwan. Commonly occurs in the mountains, being observed in lowlands only in the northern extremes of the area.

The species is characterized by conduplicate leaves with distinct (at least in the vast majority of populations) keel wing—the reason the species was originally described under *Schistochila*. Actually, leaves look supposedly smaller as the dorsal lobe is inserted into the dorsal side of stem and then on the dorsal third of the ventral lobe. The species is geographically well defined, stretching from the southern part of the Russian Far East (southward of 51° N) via the Korean Peninsula and Japan (to Kyushu). The report from Taiwan is the southernmost outpost. Kitagawa [12] (p. 291) noted this species occurs “chiefly in deciduous forests”, although in the Russian Far East, it commonly grows in hemiboreal coniferous forests as well.

***Schistochilopsisincisa*** (Schrad.) Konstant., Arctoa 3: 125, 1994.

= *Jungermannia incisa* Schrad., Syst. Samml. Crypt. Gew. 2: 5, 1797.

*= Schistochilopsis opacifolia* (Culm. ex Meyl.) Konstant., Arctoa 3: 125, 1994.

= *Lophozia opacifolia* Culm. ex Meyl., Beitr. Kryptogamenfl. Schweiz 6 (4): 174, 1924.

Descriptions in [12] (p. 287), [13] (pp. 441, 448).

**Figure 10:**
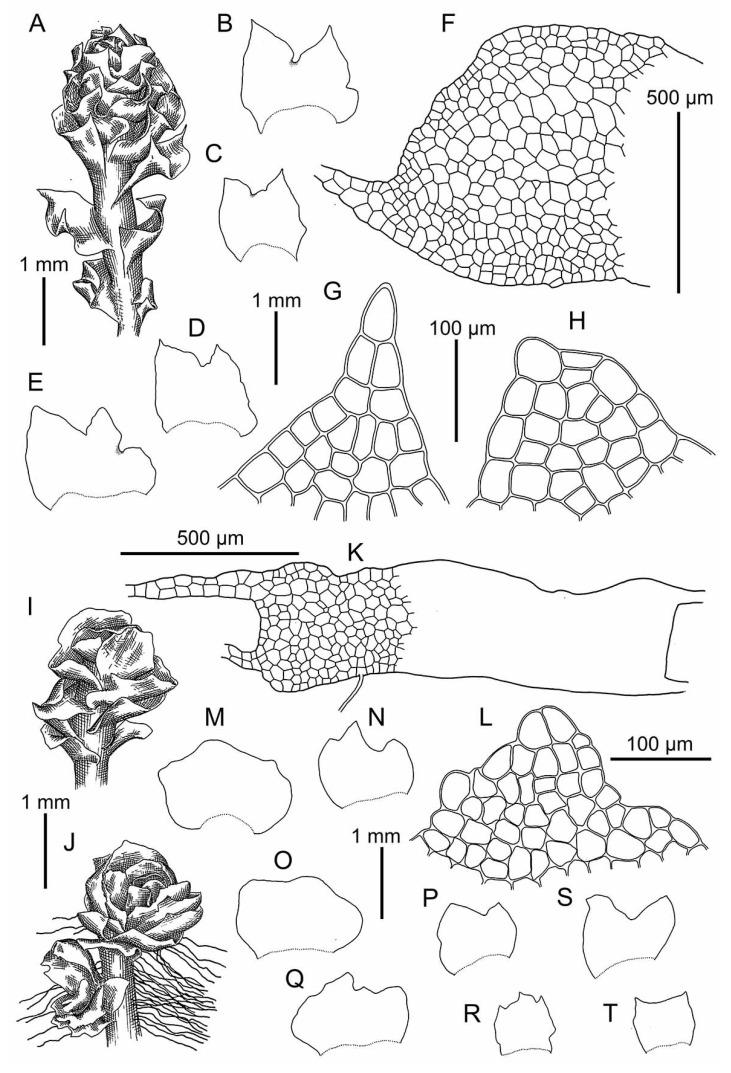
*Schistochilopsis incisa* (Schrad.) Konstant.: (**A**,**I**,**J**) Plant habit, fragment, dorsal view; (**B**–**E**,**M**–**T**) Leaves; (**G**,**H**,**L**) Cells of leaf apices; (**F**,**K**) Stem cross section with leaf base. (**A**–**H**) from K-49-20a-15 (VBGI); (**I**–**T**) from Mag-32-20-12 (VBGI).

**Figure 11:**
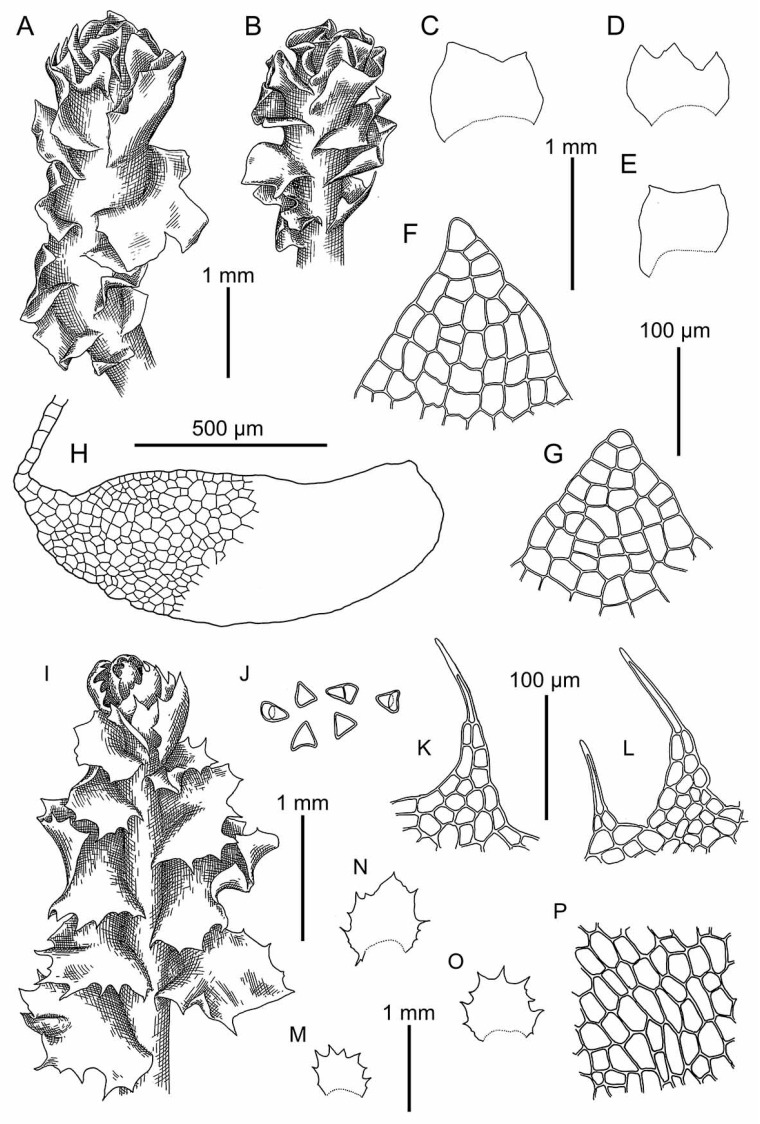
*Schistochilopsis incisa* (Schrad.) Konstant.: (**A**,**B**) Plant habit, fragment, dorsal view; (**C**–**E**) Leaves; (**F**,**G**) Cells of leaf apices; (**H**) Stem cross section with leaf base. *Schistochilopsis nakanishii* (Inoue) Konstant.: (**I**) Plant habit, fragment, dorsal view; (**J**) Gemmae; (**K**,**L**) Cells of leaf apices; (**N**–**P**) Leaves; (**P**) Leaf middle cells. (**A**–**G**) from 15-0113 (VBGI); (**I**–**P**) from Holotype TNS-174461.

Illustrations in [12] (Figure XV), [13] (Figures 173: 10–13 and 178–182), [4] (Taf. 8–13); the present paper: Figure 8B–L, Figure 9C,D, Figure 10 and Figure 11A–H.

Broadly boreo-temperate, widely spreading to northern Hemiarctic, and rarely occurring in the Arctic. The antipodal disjunction (in the Andes) was not confirmed in the present study. The occurrence of this species in India based on the report of *Schistochilopsis incisa* var. *himalayana* S. Srivast., S.C. Srivast. & K.K. Rawat (the specimens were not seen) by Srivastava et al. [48] is questionable because the taxon’s status is not clear.

This species is a very widely distributed and morphologically malleable taxon that occurs in various Northern Holarctic parts and is characterized by obliquely to substransversely inserted leaves, with prominently toothed to only scarcely crenulate margin, 1–5-stratose in lower portion leaves. The distinctions from other taxa are of quantitative and somewhat ‘negative’ nature: not so prominent teeth as in *S. setosa*, more distinct lobation in comparison with *S. nakanishii*, absence of leaf keel characterizing *S. cornuta*, not thickened outer cell wall and somewhat obliquely inserted leaves in contrast with ‘*Lophozia boliviensis*’. Despite the very large area, this species almost does not overlap the distribution of other taxa, except *S. cornuta* in East Asia, which occurs in virtually the same habitats as *S. incisa*, although commonly at lower (warmer) elevations. Southward of 35° N, the species is only revealed in the Yunnan Province of China, where its distribution is hardly expected and probably possesses relict character or is of long-dispersal origin. According to the constructed phylogenetic trees, this complex is still far from monophyly. There are different, morphologically indistinct specimens that occupy separate positions and may be regarded as distinct subspecies or even species if morphological features are found to distinguish them. At present, we are inclined to treat these deviations as the result of possible cryptic speciation and have not uncovered attempts to describe them according to morphological definitions.

The status of *Schistochilopsis incisa* var. *himalayana* is unclear. Srivastava et al. [48] (p. 143) wrote: “The variety can be differentiated from typical *S. incisa* in dorsally connated leaf near branching in robust plants (totally absent in *S. incisa*), leaf margin with few dentitions (dentitions frequent in *S. incisa*), and leaf cells without trigones (leaf cells with trigones in *S. incisa*)”. Among the listed features, connate leaves may only provide taxonomic value in the genus. Still, it is highly questionable whether this is a spontaneous aberration of a single population from a strongly shaded place (since no trigones are in the leaf cells) or the feature is really correlated with genetic differences.

***Schistochilopsisnakanishii*** (Inoue) Konstant., Arctoa 3: 125, 1994.

Basionym: *Lophozia nakanishii* Inoue, Bull. Natl. Sci. Mus. Tokyo (n.ser.) 9 (1): 37, 1966.

Holotype: Taiwan (Formosa), Nan Tow Hsien, Kuan Kao, Pa-Tung-Kuan, ca 2700 m a.s.l., 28 March 1963, S. Nakanishi, 13760, TNS-174461!

Description in [49] (p. 37), [4] (p. 132).

Illustrations in [49] (Figure 1: 10–18); present paper: Figure 11I–P.

Chiefly Taiwan endemic taxon occurring in upper elevations of mountains (known from 2700 m a.s.l.).

Söderström et al. [1] regarded this taxon as having serious doubts on the status. On the contrary, we consider it as rather good species, characterized by prominently dentate–ciliate leaves with long end cell, like that observed in *Schistochilopsis setosa*, however, different from the latter in the slightly lobed leaves as the lobation becomes not evident. The feature of small to virtually absent trigones in the leaf cells stressed by Inoue [49] may be environmentally induced.

***Schistochilopsis pacifica*** Bakalin, Botanica Pacifica 5(2): 54, 2016.

Holotype: Russia. Kamchatka Territory. East Kamchatka, Ganalsky Range, Bakening volcano area, upper course of Pravaya Kamchatka River, western slope of Bakening volcano (53°54′58′′ N 158°01′27′′ E), 1065 m alt., 6 August 2015, Vadim A. Bakalin, K-49-20-15, VBGI!

Description in [2] (p. 54).

**Figure 12:**
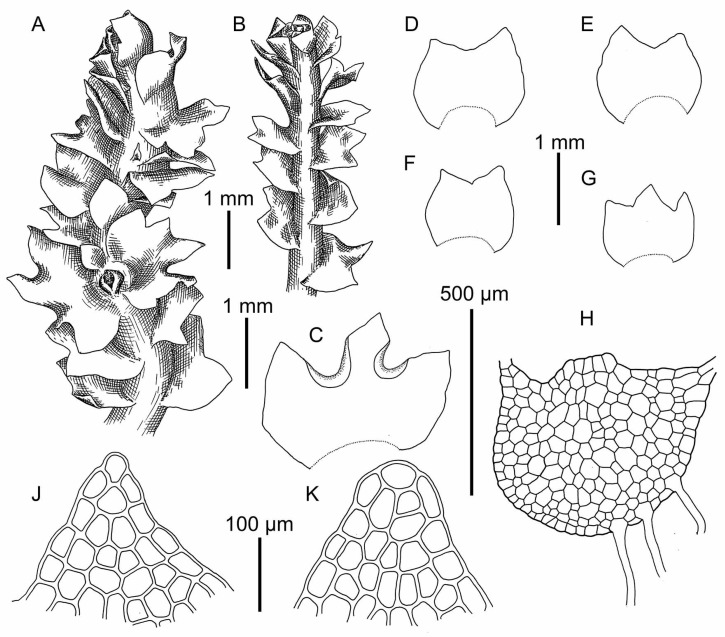
*Schistochilopsis pacifica* Bakalin: (**A**,**B**) Female plant habit, fragment, dorsal view; (**C**–**G**) Leaves; (**H**) Stem cross section with leaf bases and rhizoid bases; (**J**,**K**) Cells of leaf apices. All from K-57-11-04 (VBGI).

Illustrations in [2] (Figures 1D–F, 3 and 4A–G); present paper: Figure 9E,F and Figure 12.

Chiefly hemiarctic–boreal amphi-oceanic species known in the eastern part of Kamchatka Peninsula and southern Kuril Islands, but probably distributed wider and likely may be found in North Kurils as well as (with less probability) in upper elevations of Hokkaido.

The species is characterized by lax textured whitish plants superficially resembling ‘*Schistochilopsis opacifolia*’, from which, however, it differs in unistratose leaves, only scarcely toothed leaf margin and presence of golden-brownish pigmentation, evident at least in upper parts of subapical leaves.

***Schistochilopsis setosa*** (Mitt.) Konstant., Arctoa 3: 125, 1994.

= *Jungermannia setosa* Mitt., J. Proc. Linn. Soc., Bot. 5 (18): 92, 1860, 1861. Isotype: India, Sikkim, Hooker, no 1317; PC0102704!

= *Jungermannia pluridentata* Mitt., Journ. Proc. Linn. Soc., Bot. 5: 92.

*= Lophozia pluridentata* (Mitt.) Steph., Spec. Hep. 6: 113, 1917.

Description in [4] (p. 133).

Description based on specimens involved to the present study (Table 3 and aforementioned type) follows: plants prostrate to ascending, yellowish greenish, whitish greenish to bright green, at leaf margins sometimes discolored, soft and lax, 3–4.5 mm wide, 8–30 mm long, prostrate to ascending, in loose patches. Rhizoids numerous, forming mat under stem or obliquely spreading in unclear fascicles, colorless to pale grayish. Stem sparsely to freely laterally intercalary branched, also commonly with 1–2 subfloral innovations, cross section transversely elliptic, ca 400–600 µm × 700–800 µm, outer margin crenulate to smooth, marginal cells thin- to thick-walled, 20–32 µm in diameter, trigones virtually absent, inward 25–37(–55) µm in diameter, 4–7-gonal, trigones vestigial to small, concave, fungal infection irregular, in some cells only. Leaves contiguous, sometimes enclosed one to another, undulate, loosely concave-canaliculate, sometimes loosely recurved, obliquely inserted and oriented, not decurrent or decurrent for 1/4 of stem width dorsally, sheathing the stem in the base, when flattened in the slide ob-trapezoidal, (2–)3–4(–6)-lobed, with ventral lobe(s) larger, divided by gibbous sinus descending for 1/4–1/2 of leaf length, lower part of sinus sometimes revolute, margin commonly toothed, sometimes sparsely so, including also sinus margins, rarely margin entire, 1.0–2.5 µm × 1.2–4.0 mm. Midleaf cells 32–68 µm × (25–)32–40 µm, subisodiametric to oblong, trigones moderate, triangular to concave or convex; basal cells slightly larger, sometimes in 2 layers near very base; cells along margin 32–50 µm, external wall thin to sometimes thickened, other walls merely thin, trigones moderate to large, concave to convex; cuticle smooth throughout; marginal teeth prominently spinose, become larger to ventral base, where to 600 µm long (7 uniseriate cells), with end cell 100–180 µm long and even longer, with the 1/6–1/2 of this cell occupied by the thickened cell wall. Dioicous. Female bracts are similar to large leaves, 3–4-lobed, densely spinose. Perianth tubular, narrowed to the mouth, near mouth loosely plicate, 2–2.5 mm long and 1.2 mm in diameter; densely dentate-ciliate, commonly two cells wide at the base, terminal cell 150 µm long and even longer.

**Figure 13:**
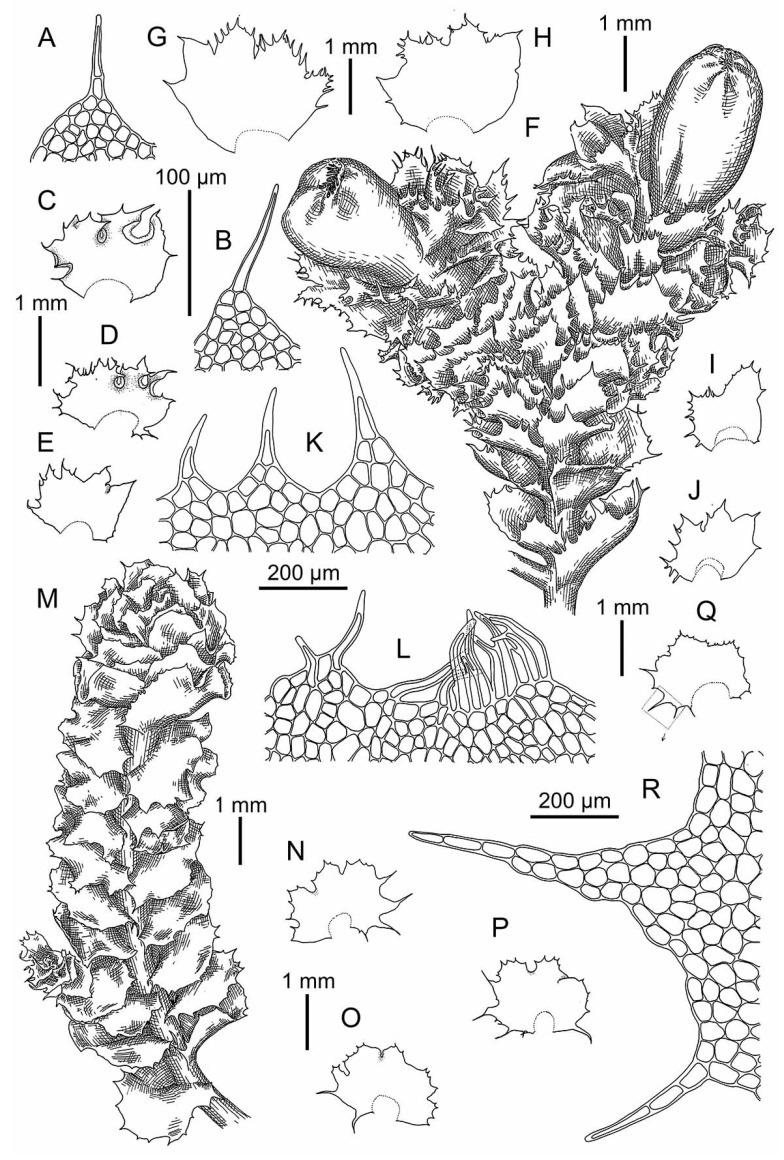
*Schistochilopsis setosa* (Mitt.) Konstant.: (**A**,**B**,**K**,**L**,**R**) Cells of leaf apices; (**C**–**E**,**G**–**J**,**N**–**Q**) Leaves; (**F**) Perianthious plant habit, fragment, dorsal view; (**M**) Plant habit, fragment, dorsal view. (**A**–**E**) from Isotype PC0102704; (**G**–**L**) from C-40-4-17 (VBGI); (**M**–**R**) from V-3-91-16 (VBGI).

Illustrations in present paper Figure 9G,H and Figure 13.

Distinctly Sino-Himalayan—Hengduan Range—North Indochinese taxon. Reported from Nepal, Indian Sikkim, Bhutan (cf. [50]), eastward widely distributed in Sichuan and Yunnan Provinces of China and uppermost elevations of Northwest Vietnam. The species should likely occur in the mountains of northern Myanmar, Thailand and Laos. In Eastern Himalaya it occurs between elevations 2900 and 5000 m a.s.l. (cf. [50]), although some previous reports may be based on misidentified *Schistochilopsis boliviensis*. The same elevations seem to be expectable in the eastern part of the species area. *Schistochilopsis setosa* distribution is restricted by the regions of monsoon climates unlike other taxa of the genus, except for *S. boliviensis*.

The species’ striking characteristics are deeply (2–)3–5(–6)-lobed and densely toothed leaves with prominently long end cell, commonly exceeding 100–120 µm long and having very thick wall at the end.

### 5.1. Doubtful Taxon

***Schistochilopsishyperarctica*** Konstant. et L.Söderstr., Phytotaxa 162 (4): 240, 2014.

Based on: *Lophozia hyperarctica* R.M.Schust., Canad. J. Bot. 39 (4): 967, 1961, *nom. inval*.

Holotype: Canada, Northwest Territories, Ellesmere Island, Alert Weather Station, F?, not seen.

Descriptions in [13] (p. 437).

Illustrations in [13] (Illustration no. 177).

Distribution unclear, known from the Canadian Arctic, Arctic Alaska, and probably North Europe [51]. All reports from Russian Asia are likely based on arctic modifications of *Schistochilopsis incisa* s.l.

Comment. The status of the taxon and its distribution are unclear. Konstantinova [51] referred it to the group of arctic species with obscure areas. Due to molecular-genetic analysis, all materials involved from North-East Asia and Siberia belong to the arctic modifications of *Schistochilopsis incisa*. The distinctions provided by Schuster [13], such as the presence of light golden pigmentation in this species, as well as wide stem with blackish colored ventral side, mainly bilobed leaves are probably environmentally induced, since the materials from the Russian Arctic and mountainous Hemiarctic are, in morphological respect, indistinguishable from the descriptions provided by Schuster [13]. Since we were unable to check the type and/or recent materials of the taxon from Arctic Canada, we cannot resolve the problem based on available materials. Bisang [4] (p. 123) also wrote on some doubts on the status of the taxon, mentioning also the necessity in fresh material study: “Im Moment kann der taxonomische Status dieser Sippe nicht beurteilt werden. Dazu muss der Typus-Beleg studiert und Frischmaterial mit Ölkörpern, die ein diagnostisches Merkmal darstellen sollen, untersucht werden”.

### 5.2. The Key

The key to *Schistochilopsis* taxa (the alternative keys to identify the vast majority of *Schistochilopsis* taxa may be found in [2,4,8,13].

1. Leaves with distinctly winged keels [hemiboreal to temperate amphi-Pacific East Asia] *Schistochilopsis cornuta*

1. Leaves without winged keels, keel absent … 2

2. Leaves only obscurely lobed, prominently dentate [Taiwan] … *Schistochilopsis nakanishii*

2. Leaves distinctly lobed, prominently dentate to nearly entire … 3

3. Arctic and mountainous northern hemiarctic species, with 2(–3)-lobed leaves, scarcely toothed or with entire leaf margin and not numerous (less 15–20) oil bodies per midleaf cell … *Schistochilopsis hyperarctica*

3. Taxa not confined to the Arctic, leaves mostly 3–5-lobed, rarely (as an exception only) bilobed, mostly densely and prominently toothed, rarely entire, oil bodies in the midleaf cells numerous, more than 20 per cell … 4

4. Plants of somewhat orthotropic growth, with leaves unistratose to the base, and golden-brownish coloration of apical part of shoots [hemiarctic-boreal amphi-Pacific] … *Schistochilopsis pacifica*

4. Plants of plagiotropic growth, rarely orthotropic, then leaves 2–5-stratose in the base or not occurring in the hemiarctic or boreal amphi-Paficic, brownish-golden coloration never developed … 5

5. Midleaf cells smaller than cells along leaf margin, trigones concave in the midleaf [Andean–Sino-Himalayan] … *Schistochilopsis boliviensis*

5. Midleaf cells larger than cells along leaf margin, trigones mostly convex in the midleaf [various areas including Sino-Himalaya] … 6

6. Leaves densely dentate-ciliate, terminal cell of the leaf lobe apices over 100 µm long, leaves mostly 3–5–lobed … *Schistochilopsis setosa*

6. Leaves dentate, but never ciliate, terminal cell of leaf lobe apices shorter 100 µm long, leaves mostly 2–3–lobed, or 4–lobed, but then terminal cell of the teeth less 50 µm long … *Schistochilopsis incisa s.l.*

### 5.3. The New Genus

***Protochilopsis*** Troizk., Bakalin et Fedosov, ***gen. nov*.**

Type species: *Jungermannia grandiretis* Lindb. ex Kaal., Nyt Mag. Naturvidensk. 33 (4/5): 322, 1893 (equivalent to *Schistochilopsis grandiretis* (Lindb. ex Kaal.) Konstant.). The genus is hitherto monotypic.

Description. Plants fleshy, whitish greenish to purplish and purple blackish, prostrate, strongly attached by the rhizoids to the substrate, acidophilic; stem transversely elliptic in the cross section, irregularly infested by fungal hyphae, ventral side purple-black, abundantly rhizogenous; leaves 3–5-lobed, lobes acute; midleaf cells large, to 80 µm × 60 µm; gemmae sporadically present, angular, with prominent angles, but not stellate.

The genus is similar to *Schistochilopsis*, from which it differs in large leaf cells and purple-black coloration of the ventral side of the stem. Hitherto, the genus is monotypic.

***Protochilopsis grandiretis*** (Lindb. ex Kaal.) Troizk., Bakalin et Fedosov, ***comb. nov.***

Basionym: *Jungermannia grandiretis* Lindb. ex Kaal., Nyt Mag. Naturvidensk. 33 (4/5): 322, 1893.

= *Schistochilopsis grandiretis* (Lindb. ex Kaal.) Konstant., Arctoa 3: 125, 1994.

Description in [13] (p. 456), [4] (p. 120).

Illustration in [13] (Figures 173: 3–5, 183 and 184); present paper: Figure 7E,F.

Generally, arctic–alpine circumpolar taxon, with the vast majority of known localities confined to Hemiarctic, where the species is mostly associated with *Sphagnum* bogs and oligotrophic mossy tundras, frequently occurs on pure peat (including degraded mossy spots). Rarely spreading to the Arctic (as far as to Spitsbergen and Northwest Greenland, Franz Josef Land, Wrangell Island) and southward to boreal and even temperate zones by alpine and subalpine belts in the mountains.

This is the only species in the genus that could be hardly mistaken with *Schistochilopsis* (as well as with other Scapaniaceae) due to peculiarly large leaf cells and purple-black coloration of the ventral side of stem (purple coloration is entirely absent in *Schistochilopsis*).

### 5.4. The Taxon Excluded From the Genus

***Lophozia obscura*** (Bakalin) Troizk., Bakalin et Fedosov, ***comb. nov.***

Basionym: *Schistochilopsis obscura* Bakalin, Bot. Pacif. 5(2): 52, 2016.

Due to data obtained in the present analysis, this specie does not belong to *Schistochilopsis* and should be transferred to *Lophozia*, where, however, likely occupies the basal position. Due to data in hand, the species is restricted to the southern Kurils (Iturup Island) where collected in a rather unique area representing the late Pleistocene caldera with modern volcanic cone where it grew just above the sulfurous steaming water or above hot stream with water strongly enriched with arsenic. This site has probably many times been disturbed in the Holocene and cannot be regarded as the only occurrence of the species that likely should be much wider distributed than it is presently known.

## Figures and Tables

**Table 1:** The climatic parameters of the main collecting areas [25].

Locality	Temperature, °C: Coldest Month Mean/Annual Mean/Warmest Month Mean	Precipitation, mm: Coldest Month Amount/Annual Amount/Warmest Month Amount	Elevation of the Weather Station	Climate Type
Northern Part of Magadan Province (based on data for Seimchan Settlement)	−36.9/−11.2/15.5	9/310/46	211	subarctic continental
Southern part of Eastern Kamchatka (based on data for Petropavlovsk-Kamchatsky)	−8.0/1.6/12.7	56/1010/144	22	subarctic oceanic
Southern Sikhote-Alin (based on data for Vladivostok)	−12.1/5.6/21.2	10/724/140	12	cool temperate monsoon
Southern Sakhalin (based on data for Yuzhno-Sakhalinsk)	−12.8/2.5/16.6	42/860/113	40	hemiboreal oceanic
Southern Kurils (based on data for Kurilsk Town)	−6.6/4.6/16.7	52/1295/172	53	hemiboreal oceanic
Western Sichuan (based on data for Xinduqiao Settlement)	−3.6/5.4/13.0	3/804/165	3472	oroboreal monsoon
Northern Yunnan (based on data for Jian-Chuan Town)	7.7/14.5/19.9	8/1029/216	2196	oroboreal monsoon
Hoang Lien Range (based on data for Sapa Town)	9.3/16.2/21.2	3/2223/467	1489	orosubtropical monsoon

**Table 2:** Primers used in the study.

Primers	Sequence 5′–3′	References
ITS-Hep4-F	CGTTGTGAGAAGTTCATTAAACC	[26]
ITS-HepD-R	CCGCYTAGTGATATGCTTAAACTC	[26]
ITS-prA-F (dir)	ACCTGCGGAAGGATCATTG	[27]
ITS-prB-R (rev)	GATATGCTTAAACTCAGCGG	[28]
trnLF-R	ATTTGAACTGGTGACACGAG	[29]
trnLC-F	CGAAATCGGTAGACGCTACG	[29]
trnGF1	ACCCGCATCGTTAGCTTG	[30]
trnGR	GCGGGTATAGTTTAGTGG	[30]

**Table 3:** The GenBank accession numbers and vouchers for those new sequence data were obtained.

ITS	trnL–trnF	trnG	Taxon	Authority	Specimen	Voucher	Origin	Collector
	MT381891	MT381854	*Schistochilopsis* cf. *hyperarctica*	Konstant. & L. Söderstr.	Mag 32-20-12	VBGI:7503	Russia: Far East, Magadan Province 61.206 N 153.898 E	V.A. Bakalin
	MT381893	MT381857	*Schistochilopsis cornuta*	(Steph.) Konstant.	K-61-4-07	VBGI:32056	Russia: Far East, Sakhalin Province 43.754 N 146.717 E	V.A. Bakalin
	MT381894	MT381858	*Schistochilopsis cornuta*	(Steph.) Konstant.	Kh-28-5-16	VBGI:19631	Russia: Far East, Khabarovsk Territory 50.306 N 134.704 E	V.A. Bakalin
	MT381895	MT381859	*Schistochilopsis cornuta*	(Steph.) Konstant.	P-16-18-12	VBGI:5551	Russia: Far East, Primorsky Territory 43.1103 N 132.7906 E	V.A. Bakalin
MT431689	MT381896	MT381860	*Schistochilopsis hyperarctica*	Konstant. & L. Söderstr.	15-0113	VBGI:32047	Russia: Krasnoyarsk Territory 69.275 N 90.012 E	V.E. Fedosov
	MT381897	MT381861	*Schistochilopsis hyperarctica*	Konstant. & L. Söderstr.	15-0436	VBGI:32049	Russia: Krasnoyarsk Territory 69.275 N 90.012 E	V.E. Fedosov
	MT381888	MT381850	*Schistochilopsis* aff. *incisa*	(Schrad.) Konstant.	Prim-62-1-17	VBGI:70516	Russia: Far East, Primorsky Territory 43.6957 N 134.20245 E	K.G. Klimova
	MT381892	MT381855	*Schistochilopsis* cf. *incisa*	(Schrad.) Konstant.	Mag-42-9-11	VBGI:14974	Russia: Far East, Magadan Province 63.256 N 151.543 E	V.A. Bakalin
MT431690	MT381898	MT381862	*Schistochilopsis incisa*	(Schrad.) Konstant.	30-15548	VBGI:32038	USA: Wyoming State 43.767 N 110.017 W	Ye.I. Kosovich-Anderson
	MT381899	MT381863	*Schistochilopsis incisa*	(Schrad.) Konstant.	Mag 33-6-14	VBGI:4644	Russia: Far East, Magadan Province 59.570278 N 150.64222 E	V.A. Bakalin
MT431691	MT381900	MT381864	*Schistochilopsis incisa*	(Schrad.) Konstant.	Mag 30-11-13	VBGI:7311	Russia: Far East, Magadan Province 59.584 N 151.142 E	V.A. Bakalin
	MT381901	MT381865	*Schistochilopsis opacifolia*	(Culm. ex Meyl.) Konstant.	15-0226	VBGI:32062	Russia: Krasnoyarsk Territory 69.275 N 90.012 E	V.E. Fedosov
	MT381902	MT381866	*Schistochilopsis opacifolia*	(Culm. ex Meyl.) Konstant.	K-49-20a-15	VBGI:3382	Russia: Far East, Kamchatka Territory 53.916 N 158.024 E	V.A. Bakalin
	MT381903	MT381867	*Schistochilopsis opacifolia*	(Culm. ex Meyl.) Konstant.	Mag 21-5-13	VBGI:7138	Russia: Far East, Magadan Province 59.799 N 149.642 E	V.A. Bakalin
MT431692	MT381904	MT381868	*Schistochilopsis opacifolia*	(Culm. ex Meyl.) Konstant.	Mag-50-7-11	VBGI:16454	Russia: Far East, Magadan Province 60.659 N 151.358 E	V.A. Bakalin
	MT381905	MT381869	*Schistochilopsis setosa*	(Mitt.) Konstant.	C-40-4-17	VBGI:37301	China: Sichuan Province 29.976 N 101.885 E	V.A. Bakalin & K.G. Klimova
	MT381906	MT381870	*Schistochilopsis setosa*	(Mitt.) Konstant.	V-3-92-16	VBGI:19211	Viet Nam: Lao Cai Province 22.303 N 103.775 E	V.A. Bakalin
		MT381871	*Schistochilopsis setosa*	(Mitt.) Konstant.	V-8-68-17	VBGI:36410	Viet Nam: Lao Cai Province 22.304 N 103.775 E	V.A. Bakalin & K.G. Klimova
	MT381890	MT381852	*Schistochilopsis setosa*	(Mitt.) Konstant.	V-3-91-16	VBGI:19209	Viet Nam: Lao Cai Province 22.303 N 103.775 E	V.A. Bakalin
		MT381853	*Schistochilopsis setosa*	(Mitt.) Konstant.	V-6-3-19	VBGI:65770	Viet Nam: Lai Châu Province 22.49992 N 103.58328 E	V.A. Bakalin & K.G. Klimova
		MT381873	*Schistochilopsis setosa*	(Mitt.) Konstant.	C-83-39-18	Personal collection: V. Bakalin	China: Yunnan Province 27.61544 N 99.89833 E	V.A. Bakalin & W.Z. Ma
MT431693	MT381910	MT381876	*Schistochilopsis pacifica*	Bakalin	K-57-11-04	VBGI:32089	Russia: Far East, Kamchatka Territory 54.814 N 167.488 E	V.A. Bakalin
	MT381911	MT381877	*Schistochilopsis pacifica*	Bakalin	K-57-9-04	VBGI:32096	Russia: Far East, Kamchatka Territory 54.814 N 167.488 E	V.A. Bakalin
MT431694	MT381912	MT381878	*Schistochilopsis pacifica*	Bakalin	K-49-20-15	VBGI:3381 Holotypus	Russia: Far East, Kamchatka Territory 53.916 N 158.024 E	V.A. Bakalin
	MT381913	MT381879	*Schistochilopsis pacifica*	Bakalin	K-76-14-15	VBGI:9014	Russia: Far East, Sakhalin Province 45.491 N 148.818 E	V.A. Bakalin
	MT381914	MT381880	*Schistochilopsis pacifica*	Bakalin	K-77-15-15	VBGI:9042	Russia: Far East, Sakhalin Province 45.496 N 148.825 E	V.A. Bakalin
	MT381915	MT381881	*Schistochilopsis pacifica*	Bakalin	K-67-4-15	VGBI:3560 Paratypus	Russia: Far East, Kamchatka Territory 53.442 N 158.652 E	V.A. Bakalin
MT431695	MT381916	MT381882	*Schistochilopsis grandiretis*	(Lindb. ex Kaal.) Konstant.	K-43-18-15	VGBI:3289	Russia: Far East, Kamchatka Territory 53.94083 N 158.02528 E	V.A. Bakalin
MT431696	MT381917	MT381883	*Schistochilopsis grandiretis*	(Lindb. ex Kaal.) Konstant.	Kh-18-19-16	VGBI:19494	Russia: Far East, Khabarovsk Territory 50.34639 N 134.65778 E	V.A. Bakalin
	MT381918	MT381884	*Schistochilopsis obscura*	Bakalin	K-79-18-15	VGBI:9121	Russia: Far East, Sakhalin Province 45.078 N 147.987 E	V.A. Bakalin
MT431697	MT381919	MT381885	*Schistochilopsis obscura*	Bakalin	K-79-21-15	VGBI:9124	Russia: Far East, Sakhalin Province 45.078 N 147.987 E	V.A. Bakalin
	MT381907	MT381872	*Schistochilopsis* sp.	(N. Kitag.) Konstant.	C-73-21a-18	Personal collection: V. Bakalin	China: Yunnan Province 26.59494 N 99.76433 E	V.A. Bakalin & W.Z. Ma
	MT381908	MT381874	*Schistochilopsis* sp.	(N. Kitag.) Konstant.	C-82-5-18	Personal collection: V. Bakalin	China: Yunnan Province 27.16517 N 100.23497 E	V.A. Bakalin & W.Z. Ma
	MT381909	MT381875	*Schistochilopsis* sp.	(N. Kitag.) Konstant.	C-82-8-18	Personal collection: V. Bakalin	China: Yunnan Province 27.16517 N 100.23497 E	V.A. Bakalin & W.Z. Ma
	MT381887	MT381849	*Schistochilopsis* sp. *(sichuanica)*	(N. Kitag.) Konstant.	C-39-7-17	VBGI:37288	China: Sichuan Province 29.977 N 101.885 E	V.A. Bakalin & K.G. Klimova
	MT381889	MT381851	*Schistochilopsis* sp.	(N. Kitag.) Konstant.	C-35-3-17	VBGI:37439	China: Sichuan Province 29.991 N 101.888 E	V.A. Bakalin & K.G. Klimova
	MT381920	MT381886	*Schistochilopsis* sp.	(N. Kitag.) Konstant.	VF92a	VGBI:49423	Russia: Krasnoyarsk Territory 73.48427 N 80.57096 E	V.E. Fedosov
